# Serum Autotaxin Levels Predict Liver-Related Events in Patients With Primary Biliary Cholangitis: A Long-Term Multicenter Observational Study

**DOI:** 10.14309/ctg.0000000000000779

**Published:** 2024-10-17

**Authors:** Takanobu Iwadare, Takefumi Kimura, Yuki Yamashita, Taiki Okumura, Shun-ichi Wakabayashi, Hiroyuki Kobayashi, Ayumi Sugiura, Tomoo Yamazaki, Satoshi Shimamoto, Koji Igarashi, Satoru Joshita, Takeji Umemura

**Affiliations:** 1 Department of Medicine, Division of Gastroenterology and Hepatology, Shinshu University School of Medicine, Matsumoto, Japan;; 2 Consultation Center for Liver Diseases, Shinshu University Hospital, Matsumoto, Japan;; 3 Department of Internal Medicine, Sato Hospital, Nakano, Japan;; 4 Department of Medicine, University of California San Diego, La Jolla, USA;; 5 Bioscience Division, TOSOH Corporation, Ayase, Japan;; 6 Department of Medicine, Yodakubo Hospital, Nagawa, Japan.

**Keywords:** autotaxin, primary biliary cholangitis, liver-related events, hepatocellular carcinoma, biomarker, prognosis

## Abstract

**INTRODUCTION:**

A straightforward, reliable, and noninvasive method for predicting the development of liver-related events (LRE) in primary biliary cholangitis (PBC) has not been attained thus far. This study investigated whether serum autotaxin (ATX) could predict LRE in patients with PBC.

**METHODS:**

This retrospective multicenter investigation included 190 biopsy-proven untreated patients with PBC. All subjects were followed for at least 1 year, during which time the prevalence of LRE, including newly developing hepatocellular carcinoma, esophagogastric varices, ascites, and hepatic encephalopathy, was investigated in relation with ATX levels at the time of liver biopsy.

**RESULTS:**

During the median follow-up period of 9.7 years, LRE were observed in 22 patients (11.6%). The area under the receiver operating characteristic curve and cutoff value of serum ATX for predicting LRE were 0.80 and 1.086 mg/L, respectively. Patients with serum ATX ≥1.086 had a significantly higher cumulative incidence of LRE compared with patients with ATX < 1.086 (33.3% vs 3.6%, *P* < 0.00001). Notably, the predictive capability of ATX for LRE in patients with PBC surpassed that of FIB-4, ALBI, APRI, and Mac-2-binding protein glycan isomer. A multivariate Cox proportional hazards model revealed ATX as an independent associated factor for LRE (hazard ratio 6.24, 95% confidence interval 1.87–20.80, *P* = 0.003) along with Nakanuma stage (hazard ratio 2.75, 95% confidence interval 1.52–4.99, *P* < 0.001). These results were closely replicated in a serologically diagnosed PBC validation cohort (n = 32).

**DISCUSSION:**

Serum ATX levels may serve as a predictive marker for LRE in patients with PBC.

## Graphical abstract

**Figure:**
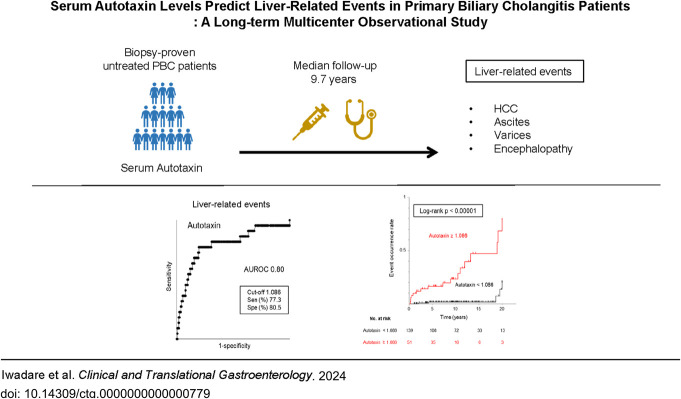

## INTRODUCTION

Primary biliary cholangitis (PBC) is an autoimmune liver disorder that is characterized by a female predominance, chronic and destructive clinical course, small bile duct, granulomatous lymphocytic cholangitis, and typical seroreactivity for anti-mitochondrial antibodies (AMA) (1). Ursodeoxycholic acid (UDCA) is the most effective treatment of PBC and significantly improves disease prognosis regardless of the disease stage and observed biochemical response (2). Nevertheless, some patients progress to cirrhosis, liver failure, esophagogastric varices, and hepatocellular carcinoma (HCC) (3).

Several factors have been identified as indicators of a poorer prognosis in PBC, including the presence of symptoms at diagnosis, resistance to UDCA therapy, more advanced histological staging, and such prognostic scores as fibrosis-4 index (FIB-4), albumin-bilirubin score (ALBI), and aspartate aminotransferase (AST)-to-platelet ratio index (APRI) (4–7). While a liver biopsy, which accurately assesses the clinical stage of PBC, can provide valuable insights into the extent of inflammatory activity and liver fibrosis, its use is often constrained by invasiveness, discomfort, potential sampling error, and interobserver variability (8). Currently, the achievement of straightforward, dependable, and noninvasive methods capable of predicting the prognosis of PBC remains unrealized.

Autotaxin (ATX) was originally discovered in human melanoma cell cultures (9). The protein is encoded by the ectonucleotide pyrophosphatase/phosphodiesterase family member 2 gene, which catalyzes the hydrolysis of lysophosphatidylcholine to lysophosphatidic acid (LPA) and functions as a phospholipase (10). We have reported that serum ATX levels correlate with liver inflammation activity and fibrosis severity in viral hepatitis, PBC, and metabolic dysfunction-associated steatotic liver disease (MASLD) (11–15).

Serum ATX measurement has been covered by Japanese national health insurance for patients with chronic hepatitis and liver cirrhosis since 2018, and its clinical application is already established. However, to the best of knowledge, no studies have evaluated the significance of ATX as a predictor of liver-related PBC outcome. Here, we showed circulating ATX levels reflected the risk of liver-related events (LRE) in untreated patients with PBC.

## METHODS

### Patients and clinical examinations

This retrospective multicenter study was approved by the Committee for Medical Ethics of Shinshu University School of Medicine (ID number: 3244) and performed in accordance with the Declaration of Helsinki 1975, 1983 revision. We firstly enrolled 190 Japanese patients with biopsy-proven PBC followed for at least 1 year at 9 medical institutions across Nagano Prefecture (Shinshu University Hospital, Nagano Red Cross Hospital, Azumino Red Cross Hospital, Nagano Municipal Hospital, Iida Municipal Hospital, Aizawa Hospital, Showa Inan General Hospital, Maruko Central Hospital, and Fujimori Hospital) between July 1992 and February 2021. The diagnosis of PBC was based on criteria established by the Intractable Hepato-Biliary Diseases Study Group of Japan (16). Serum AMA specific for the pyruvate dehydrogenase complex-E2 component were measured by the enzyme-linked immunosorbent assay, for which >7.0 U/mL was considered a positive result. No patient had the concurrent use of immunomodulatory drugs or corticosteroids, and none were coinfected with the hepatitis C virus or hepatitis B virus or exhibited evidence of alcoholic liver disease or MASLD. All patients were naive for UDCA treatment, with UDCA (13–15 mg/kg/d) only being prescribed after diagnosis. In addition, we recruited a validation cohort of 32 Japanese patients with PBC seen at 3 medical institutions in Nagano Prefecture (Shinshu University Hospital, Azumino Red Cross Hospital, and Nagano Municipal Hospital) between November 1991 and February 2023, in whom PBC was diagnosed serologically without biopsy, serum was available before UDCA treatment, and follow-up was continued for at least 1 year. All laboratory data were obtained in an overnight fasting state on the day of liver biopsy. FIB-4, ALBI, and APRI were calculated according to the following formulas: FIB-4 = (age [years] × AST [U/L])/(platelet count (Plt) [×10^9^/L] × alanine aminotransferase (ALT) [U/L]^1/2^), ALBI score = (log_10_ total bilirubin (T-bil) [mg/dL] × 17.1 × 0.66) + (albumin [g/dL] × 10 × −0.085), and APRI = AST [U/L]/upper limit of normal range [U/L]/Plt [10^9^/L] × 100 (17–19).

The diagnosis of autoimmune hepatitis (AIH)-PBC overlap syndrome required the presence of at least 2 of 3 diagnostic criteria for each disease. For PBC, these criteria included serum alkaline phosphatase (ALP) levels at least twice the upper limit of normal or serum γ-glutamyltranspeptidase (γ-GT) levels at least 5 times the upper limit of normal, a positive test for AMA, and a liver biopsy specimen showing florid bile duct lesions. For AIH, the criteria included serum ALT level at least 5 times the upper limit of normal, serum IgG level, at least twice the upper limit of normal or a positive test for anti-smooth muscle antibodies, and liver biopsy showing moderate or severe periportal or periseptal lymphocytic piecemeal necrosis (20).

ATX was measured using serum samples obtained at the time of liver biopsy. Serum ATX concentrations were determined using a 2-site immunoenzymetric assay with the TOSOH AIA system (TOSOH, Tokyo, Japan) (21). This study used a solid-phase antibody produced by a rat antihuman ATX monoclonal antibody-producing cell clone, designated as R10.23, and a labeled antibody using the clone designated as R10.21 (22).

### Histological examinations

All pathological examinations were performed at Shinshu University. Liver specimens of at least 1.5 cm in length were obtained from segments 5 or 8 using a 14-gauge needle as described previously and then immediately fixed in 10% neutral formalin. Sections of 4 μm in thickness were cut and stained using the hematoxylin and eosin and Azan-Mallory methods. The histological activity of PBC was independently assessed by an expert pathologist in a blinded manner. Disease stage was determined according to the Nakanuma classification (23).

### Patient follow-up

Analyzed patients were monitored at their respective hospital every 12 months by ultrasonography or computed tomography in addition to measurement of serum alpha-fetoprotein, with cirrhotic patients evaluated at least every 6 months (24). The radiological diagnosis of HCC was based on the American Association for the Study of Liver Diseases practice guidelines on the management of HCC as either (i) the presence of a hepatic lesion >2 cm in diameter with a typical vascular pattern for HCC on 1 dynamic imaging technique or alpha-fetoprotein >200 ng/mL or (ii) the presence of a lesion 1–2 cm in diameter with a typical vascular pattern for HCC on 2 dynamic imaging techniques (25). For patients with known cirrhosis and a Mayo risk score >4.1, upper endoscopy to assess for varices was performed at least every 2–3 years (24).

LRE were defined as the development of HCC, hepatic encephalopathy of grade II or higher, poorly controlled ascites requiring hospitalization, and esophagogastric varices necessitating endoscopic ligation, sclerotherapy, or balloon-occluded retrograde transvenous obliteration, including varices rupture. Follow-up time was defined as the number of years from diagnosis of PBC to event occurrence or from diagnosis of PBC to the last follow-up visit when protocol surveillance confirmed no event.

### Statistical analysis

Clinical data were expressed as the number (percentage) or as the median (interquartile range [IQR]). Statistical analyses were performed using StatFlex Ver. 7.0 software (Artech, Osaka, Japan) and R software ver. 4.3.0. The Mann-Whitney *U* test, χ^2^ test, and Fisher exact test were used for comparisons between study groups. Diagnostic accuracy was evaluated using the area under the receiver operating characteristic curve (AUROC). The Youden index identified cutoff values, with the nearest clinically applicable value to the cutoff considered the optimal threshold for clinical convenience. The Kaplan-Meier method, log-rank testing, and Harrell c-index were used to assess disease progression. The Cox proportional hazards model was adopted to examine univariate and multivariate covariates for LRE. All statistical tests were 2-tailed and evaluated at the 0.05 level of significance.

## RESULTS

### Baseline characteristics

The clinicopathological features of the 190 patients with biopsy-proven PBC who were monitored for at least 1 year are presented in Table 1. The median age at the time of biopsy was 59 years, and 31 patients (16.3%) were male. Pruritus was reported in 4 of 176 patients (2.3%) with documented symptoms. Antinuclear antibody (ANA) was positive in 133 of 190 patients (70%). The ANA patterns observed were as follows: homogeneous pattern in 21 patients, speckled pattern in 49 patients, discrete speckled pattern in 49 patients, nuclear envelope pattern in 15 patients, cytoplasmic pattern in 31 patients, and other in 13 patients. AIH-PBC overlap syndrome was diagnosed in 7 of 177 cases (3.9%) with complete diagnostic data available. The median values for T-bil, AST, ALT, ALP, and γ-GT were 0.8 mg/dL, 40 U/L, 41 U/L, 149 U/L, and 126 U/L, respectively. The median prognostic score values for FIB-4, ALBI score, and APRI were 1.6, −2.88, 0.48, respectively. The respective values for median serum ATX and Mac-2-binding protein glycan isomer (M2BPGi) were 0.90 mg/L and 0.86. The histopathological classification by Nakanuma stage 0/1/2/3/4 was 1/95/61/28/5 patients, respectively.

**Table 1.** Clinical characteristics of patients with biopsy-proven PBC

	Median (IQR)/n (%)
Age (yr)	59 (51–66)
Male	31 (16.3)
Pruritus^a^	4 (2.3)
ANA positive^d^	133 (70)
ANA pattern^b^ (homogenous/speckled/discrete speckled/nuclear envelope/cytoplasmic/others/ND)	21/49/49/15/31/13/9
AIH-PBC overlap syndrome^c^	7 (3.9)
Albumin (g/dL)	4.2 (4.0–4.4)
T-bil (mg/dL)	0.8 (0.6–1.0)
AST (U/L)	40 (28–64)
ALT (U/L)	41 (26–72)
ALP (U/L)	149 (106–212)
γ-GT (U/L)	126 (72–247)
TC (mg/dL)	218 (185–241)
TG (mg/dL)	99 (74–142)
LDL-C (mg/dL)	121 (104–139)
HDL-C (mg/dL)	61 (49–73)
Plt (×10^4^/μL)	22.9 (18.7–26.5)
HbA1c (%)	5.5 (5.2–5.8)
FIB-4	1.6 (1.2–2.4)
ALBI	−2.88 (−3.03 to −2.65)
APRI	0.48 (0.32–0.74)
M2BPGi^d^	0.86 (0.53–1.41)
ATX (mg/L)	0.90 (0.76–1.11)
Nakanuma stage (0/1/2/3/4)	1/95/61/28/5 (1/50/32/15/3)

AIH, autoimmune hepatitis; ALBI, albumin-bilirubin score; ALT, alanine aminotransferase; ALP, alkaline phosphatase; ANA, antinuclear antibody; APRI, aspartate aminotransferase-to-platelet ratio index; AST, aspartate aminotransferase; ATX, autotaxin; FIB-4, fibrosis-4 index; γ-GT, gamma-glutamyltransferase; HbA1c, hemoglobin A1c; HDL-C, high density lipoprotein cholesterol; IQR, interquartile range; LDL-C, low density lipoprotein cholesterol; M2BPGi, Mac-2-binding protein glycan isomer; ND, no data (ANA positive, but pattern unknown); PBC, primary biliary cholangitis; Plt, platelet count; T-bil, total bilirubin; TC, total cholesterol; TG, triglycerides.

a n = 176.

b Included multiple patterns in a case.

c n = 177.

d n = 124.

Interestingly, patients with pruritus had higher serum ATX levels compared with the group without pruritus (*P* = 0.032) (see Supplementary Figure 1, http://links.lww.com/CTG/B213). In this cohort, the presence or absence of ANA, along with specific ANA patterns, demonstrated no statistically significant association with serum ATX levels (ANA *P* = 0.203; homogeneous pattern *P* = 0.202; speckled pattern *P* = 0.451; discrete speckled pattern *P* = 0.128; nuclear envelope pattern *P* = 0.949; cytoplasmic pattern *P* = 0.109) (see Supplementary Figure 2, http://links.lww.com/CTG/B213)

### Occurrence of events

The median follow-up evaluation period for the 190 patients was 9.7 years (IQR: 5.6–14.4 years) (Table 2). Nineteen patients reached death (16 [8.4%] patients, 3 male and 13 female) or liver transplantation (3 [1.6%] patients, all female). Eight deaths were related to liver issues, with the remainder due to interstitial pneumonia, cerebral hemorrhage, and an unknown cause in 1, 1, and 6 patients, respectively. Twenty-two patients (11.6%) newly exhibited LRE of HCC, esophagogastric varices, ascites, and hepatic encephalopathy in 8, 15, 11, and 6 patients, respectively, including multiple LRE detected simultaneously. The LRE comprised 4 cases of HCC, 11 cases of esophagogastric varices, 11 cases of ascites, and 6 cases of hepatic encephalopathy.

**Table 2.** Events during follow-up

	All (n = 190)
	Median (IQR)/n (%)
Follow-up (yr)	9.7 (5.6–14.4)
Events during follow-up	
Death or liver transplantation	19 (10)
Death	16 (8.4)
Liver-related death	8 (4.2)
Liver transplantation	3 (1.6)
LRE^a^	22 (11.6)
HCC	8 (4.2)
Esophagogastric varices	15 (7.9)
Ascites	11 (5.8)
Hepatic encephalopathy	6 (3.2)

HCC, hepatocellular carcinoma; IQR, interquartile range; LRE, liver-related events.

a Including 13 patients in whom multiple LRE were found simultaneously: 1 case of HCC + ascites, 5 cases of varices + ascites, 1 case of varices + encephalopathy, 1 case of ascites + encephalopathy, 1 case of HCC + varices + ascites, 2 cases of varices + ascites + encephalopathy, 1 case of HCC + varices + encephalopathy, and 1 case of HCC + varices + ascites + encephalopathy.

### Comparison of clinicopathological features between non-LRE and LRE patients

To identify the predictors of LRE, clinicopathological features at the time of biopsy were compared between non-LRE and LRE patients (Table 3). Patients with LRE tended to have more pruritic symptoms (*P* = 0.053) and a higher prevalence of positive ANA (*P* = 0.078) compared with non-LRE patients; however, the differences did not reach statistical significance in this cohort (26,27). Patients with LRE had significantly higher levels of T-bil (*P* = 0.047), FIB-4 (*P* = 0.005), ALBI (*P* = 0.010), APRI (*P* < 0.001), and ATX (*P* < 0.001), as well as lower levels of albumin (*P* = 0.002) and hemoglobin A1c (*P* = 0.007), vs non-LRE patients. Regarding pathological findings, patients with LRE had a significantly higher Nakanuma stage than non-LRE patients (*P* < 0.001).

**Table 3.** Comparison of clinicopathological features at time of biopsy between non-LRE and LRE patients

	Non-LRE (n = 168)	LRE (n = 22)	*P* value
	Median (IQR)/n (%)	Median (IQR)/n (%)	
Age (yr)	59 (52–66)	59 (49–67)	0.906
Male	27 (16)	4 (18)	0.763
Pruritus^a^	2 (1)	2 (11)	0.053
ANA positive^d^	58 (35)	17 (77)	0.078
AIH-PBC overlap syndrome^b^	7 (4)	0 (0)	1.000
Albumin (g/dL)	4.2 (4.0–4.4)	4.1 (3.7–4.3)	**0.002**
T-bil (mg/dL)	0.8 (0.6–1.0)	0.9 (0.7–1.2)	**0.047**
AST (U/L)	38 (28–58)	70 (50–95)	0.088
ALT (U/L)	38 (25–70)	65 (42–91)	0.517
ALP (U/L)	147 (105–204)	192 (121–336)	0.087
γ-GT (U/L)	124 (72–230)	175 (81–466)	0.065
TC (mg/dL)	217 (188–236)	228 (168–267)	0.283
TG (mg/dL)	99 (72–143)	105 (89–129)	0.945
LDL-C (mg/dL)	120 (104–136)	132 (109–159)	0.360
HDL-C (mg/dL)	61 (51–72)	59 (32–78)	0.243
Plt (×10^4^/μL)	23.0 (18.7–26.7)	21.1 (15.4–24.8)	0.145
HbA1c (%)	5.5 (5.3–5.9)	5.2 (4.8–5.5)	**0.007**
FIB-4	1.6 (1.18–2.27)	2.2(1.6–5.1)	**0.005**
ALBI	−2.89 (−3.03 to −2.68)	−2.57(−2.93 to −2.29)	**0.010**
APRI	0.45 (0.30–0.69)	0.87 (0.56–5.05)	**<0.001**
M2BPGi^c^	0.84 (0.52–1.2)	1.62 (0.75–3.39)	0.092
ATX (mg/L)	0.88 (0.74–1.03)	1.36 (1.10–1.78)	**<0.001**
Nakanuma stage (0/1/2/3/4)	1/89/57/21/0 (1/53/34/12/0)	0/6/4/7/5 (0/27/18/32/23)	**<0.001**

ALBI, albumin-bilirubin score; ALT, alanine aminotransferase; ALP, alkaline phosphatase; APRI, aspartate aminotransferase-to-platelet ratio index; AST, aspartate aminotransferase; ATX, autotaxin; FIB-4, fibrosis-4 index; γ-GT, gamma-glutamyltransferase; HbA1c, hemoglobin A1c; HDL-C, high density lipoprotein cholesterol; IQR, interquartile range; LDL-C, low density lipoprotein cholesterol; LRE, liver-related events; M2BPGi, Mac-2-binding protein glycan isomer; Plt, platelet count; T-bil, total bilirubin; TC, total cholesterol; TG, triglycerides.

a n = 176, the Fisher exact test was used.

b n = 177, the Fisher exact test was used.

c n = 124.

d The Fisher exact test was used.

Bold entries indicate *P* < 0.05.

### Cumulative event incidence rate by ATX

**Figure 1.**
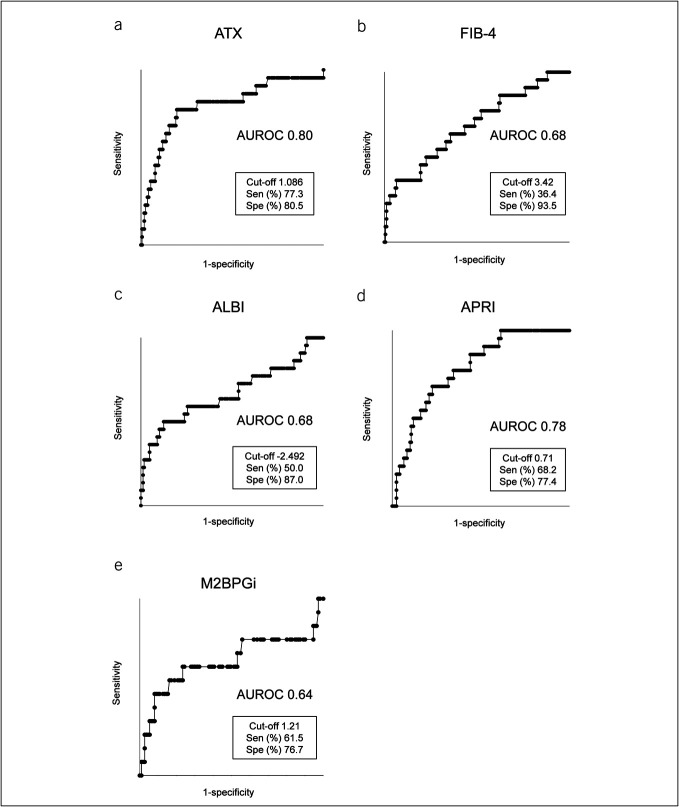
Receiver operating characteristic analysis of serum ATX levels and other prognostic scores. (**a–e**) Receiver operating characteristic analysis of ATX (**a**), FIB-4 index (**b**), ALBI (**c**), APRI (**d**), and M2BPGi (**e**) for LRE. ALBI, and albumin-bilirubin score; APRI, aspartate aminotransferase-to-platelet ratio index; ATX, autotaxin; AUROC, area under the receiver operating characteristic curve; FIB-4, fibrosis-4 index; LRE, liver-related events; M2BPGi, Mac-2-binding protein glycan isomer; Sen, sensitivity; Spe, specificity.

**Figure 2.**
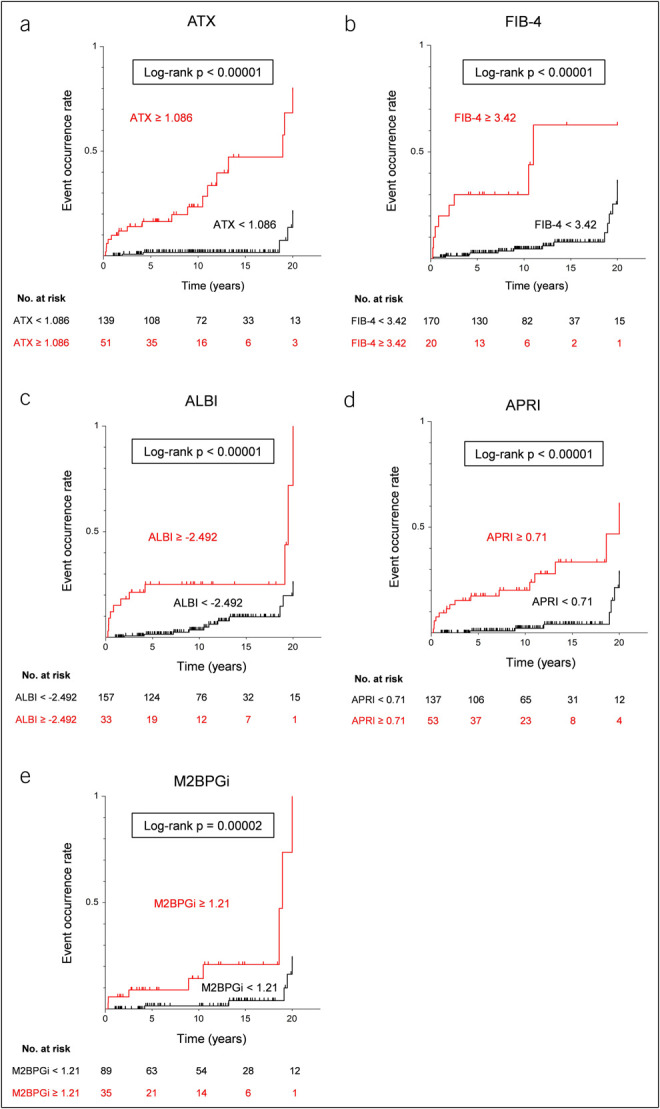
Cumulative event incidence rate analysis of serum ATX levels and other prognostic scores. (**a–e**) Cumulative event incidence rate analysis by the Kaplan-Meier method of ATX (**a**), FIB-4 (**b**), ALBI (**c**), APRI (**d**), and M2BPGi (**e**) for LRE. ATX, autotaxin; ALBI, albumin-bilirubin score; APRI, aspartate aminotransferase-to-platelet ratio index; FIB-4, fibrosis-4 index; LRE, liver-related events; M2BPGi, Mac-2-binding protein glycan isomer.

The AUROC for the prediction of LRE occurrence was high at 0.80 (Figure 1a). The identified cutoff value was 1.086 mg/L, yielding a sensitivity of 77.3% and specificity of 80.5%. Using this cutoff value, the cumulative LRE incidence rate was significantly greater in high-ATX patients (log rank *P* < 0.00001) (Figure 2a). Even after excluding AIH-PBC overlap syndrome cases, the incidence of LRE remained significantly higher in high-ATX patients compared with low-ATX patients (log-rank *P* < 0.00001) (see Supplementary Figure 3, http://links.lww.com/CTG/B213). These results indicated that serum ATX levels at the time of diagnosis could be a useful parameter for predicting the occurrence of LRE.

### Cumulative event incidence rates by conventional prognostic scores

The AUROC values for predicting LRE were 0.68 for FIB-4, 0.68 for ALBI, 0.78 for APRI, and 0.64 for M2BPGi, with respective cutoff values of 3.42, −2.492, 0.71, and 1.21 (Figure 1b–e). The sensitivity and specificity at each cutoff value were 36.4% and 93.5% for FIB-4, 50.0% and 87.0% for ALBI, 68.2% and 77.4% for APRI, and 61.5% and 76.7% for M2BPGi.

Kaplan-Meier analysis was conducted for the LRE and non-LRE occurrence groups next. Factors associated with the higher LRE occurrence groups included FIB-4 ≥ 3.42 (*P* < 0.00001), ALBI ≥ −2.492 (*P* < 0.00001), APRI ≥ 0.71 (*P* < 0.00001), and M2BPGi ≥ 1.21 (*P* = 0.00002) (Figure 2b–e).

### Validation cohort verification results

The validation cohort consisted of 32 untreated patients with a serological diagnosis of PBC, whose clinicopathological features are presented in Supplementary Digital Content (see Supplementary Table 1, http://links.lww.com/CTG/B214). The median age at the time of ATX measurement was 63 years, and 10 patients (31%) were male. The median values for T-bil, AST, ALT, ALP, and γ-GT were 0.7 mg/dL, 35 U/L, 32 U/L, 124 U/L and 111 U/L, respectively. The median serum ATX was 0.81 mg/dL. The median follow-up evaluation period was 5.3 years (IQR: 3.1–10.3 years) (see Supplementary Table 2, http://links.lww.com/CTG/B214). LRE were observed in 4 patients, including 2 patients in whom multiple LRE were found simultaneously, comprising 1 case of HCC, 2 cases of esophagogastric varices, 2 cases of ascites, and 2 cases of encephalopathy, including those detected simultaneously, comprised 1 cases of HCC, 1 case of esophagogastric varices, 2 cases of ascites, and 1 cases of hepatic encephalopathy. Patients with LRE had significantly higher levels of ATX (*P* = 0.007) compared with non-LRE patients, with no significant differences observed for other parameters (see Supplementary Table 3, http://links.lww.com/CTG/B214).

**Figure 3.**
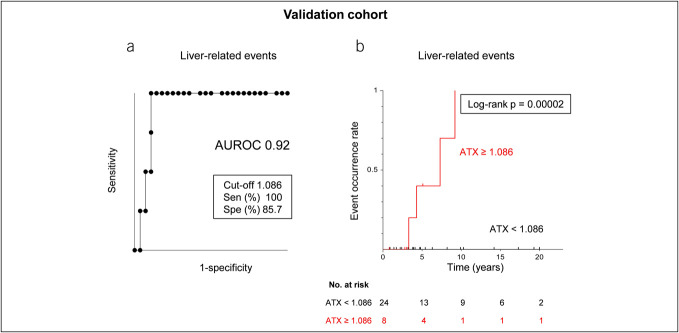
Receiver operating characteristic analysis and cumulative event incidence rate analysis of serum ATX levels for LRE in validation cohort. (**a, b**) Receiver operating characteristic analysis (**a**) and cumulative event incidence rate analysis by the Kaplan-Meier method (**b**) of serum ATX levels for LRE in the validation cohort. ATX, autotaxin; AUROC, area under the receiver operating characteristic curve; LRE, liver-related events; Sen, sensitivity; Spe, specificity.

The AUROC for ATX in the context of LRE was notably high at 0.92 (Figure 3a). Evaluation of LRE screening performance in the liver biopsy cohort using the earlier determined ATX cutoff value of 1.086 (Figure 1a) demonstrated high accuracy, with a sensitivity of 100% and specificity of 85.7%. Kaplan-Meier survival analysis using this cutoff revealed a significantly elevated rate of LRE onset in the high-ATX subgroup (log-rank *P* = 0.00002) (Figure 3b). Thus, the validation strongly further supported the efficacy of ATX as a predictor of LRE development.

### Univariate and multivariate Cox proportional hazards models for LRE risk determination

In univariate Cox proportional hazards testing, age (≥65 years) (hazard ratio [HR] 1.92, 95% confidence interval [CI] 0.76–4.86; *P* = 0.200, c-index 0.573), gender (female) (HR 0.56, 95% CI 0.19–1.71; *P* = 0.300, c-index 0.549), FIB-4 (≥3.42) (HR 7.73, 95% CI 3.20–18.68; *P* < 0.001, c-index 0.723), ALBI (≥−2.492) (HR 6.38, 95% CI 2.72–14.97; *P* < 0.001, c-index 0.741), M2BPGi (≥1.21) (HR 9.77, 95% CI 2.96–32.12; *P* < 0.001, c-index 0.762), APRI (≥0.71) (HR 6.49, 95% CI 2.64–15.93; *P* < 0.001, c-index 0.788), Nakanuma stage (HR 4.27, 95% CI 2.62–6.97; *P* < 0.001, c-index 0.778), and ATX (≥1.086) (HR 12.56, 95% CI 4.61–34.21; *P* < 0.001, c-index 0.806) were identified as determinants of LRE (see Supplementary Table 4, http://links.lww.com/CTG/B214). A noteworthy discovery was that the c-index of ATX outperformed the conventional prognostic scores for LRE.

In multivariate model 1 incorporating age, gender, and APRI having the highest c-index among the conventional prognostic scores for LRE in univariate analysis, APRI and ATX were significant factors associated with LRE (APRI: HR 3.52, 95% CI 1.34–9.24, *P* = 0.010; ATX: HR 10.12, 95% CI 3.46–29.65, *P* < 0.001) (c-index 0.903) (Table 4). Multivariate model 2 considered age, gender, and ATX alongside the histological factor of Nakanuma stage. In this model, Nakanuma stage and ATX emerged as factors significantly associated with LRE (Nakanuma stage: HR 2.75, 95% CI 1.52–4.99, *P* < 0.001; ATX: HR 6.24, 95% CI 1.87–20.8, *P* = 0.003) (c-index 0.876). Notably, the results of both multivariate models, which showed high c-indexes, supported ATX as an independent factor contributing to the occurrence of LRE in patients with PBC.

**Table 4.** Factors associated with LRE in PBC

	Univariate			Multivariate model 1			Multivariate model 2		
	HR	95% CI of HR	*P* value	HR	95% CI of HR	*P* value	HR	95% CI of HR	*P* value
Age (≥65 yr)	1.92	0.76–4.86	0.200	2.39	0.82–6.95	0.110	2.54	0.85–7.60	0.095
Gender (female)	0.56	0.19–1.71	0.300	0.39	0.12–1.31	0.127	0.37	0.10–1.33	0.128
APRI (≥0.71)	6.49	2.64–15.93	**< 0.001**	3.52	1.34–9.24	**0.010**	—	—	—
Nakanuma stage	4.27	2.62–6.97	**< 0.001**	—	—	—	2.75	1.52–4.99	**<0.001**
ATX (≥1.086)	12.56	4.61–34.21	**< 0.001**	10.12	3.46–29.65	**< 0.001**	6.24	1.87–20.8	**0.003**

APRI, aspartate aminotransferase-to-platelet ratio index; ATX, autotaxin; CI, confidence interval; HR, hazard ratio; LRE, liver-related events; PBC, primary biliary cholangitis.

## DISCUSSION

### Main findings

This study evaluated serum ATX in patients with PBC to determine its potential to estimate the development of LRE. Receiver operating characteristic analysis revealed a high AUROC value of 0.80 for LRE prediction, with log-rank analysis displaying a high cumulative LRE incidence rate (*P* < 0.00001). Compared with the conventional prognostic scores for FIB-4, ALBI, APRI, and M2BPGi, ATX was a superior indicator based on c-index results (5–7). To our knowledge, this is the first report evaluating serum ATX and LRE development in PBC. It is also noteworthy that the prognostic ability of LRE is independent of, and exceeds, that of pathological data.

### Context with published literature

ATX has been identified as a novel contributor to the pathogenesis of liver fibrosis (28,29). According to Ikeda et al, LPA, which is produced by ATX-mediated responses, stimulates the proliferation and contractility of hepatic stellate cells, the main producers of extracellular matrix components in the liver (30,31). Another indirect theory exists regarding the relationship between serum ATX and liver fibrosis. In the healthy liver, sinusoidal endothelial cells clear ATX taken up from hepatic sinusoids. In fibrotic liver tissue, however, the capillarization of sinusoids leads to reduced ATX uptake and increased plasma ATX levels (32). Thus, any liver condition that causes fibrosis may result in elevated plasma ATX.

In previous research, the ATX-LPA signaling axis was shown to play an important role in both normal physiology and disease pathogenesis and was linked to pruritus in chronic cholestatic liver diseases, including PBC (33). A noteworthy association between serum ATX levels and pathological disease progression, severity of liver injury, and overall survival in PBC has been established (14,34,35). Our discovery that ATX values can independently predict LRE regardless of liver pathological stage is particularly significant. Further investigations are required to elucidate the mechanisms underlying the relationship between ATX and LRE development.

In MASLD, ATX secreted by hepatocytes exacerbated disease pathology by autocrine inhibition of peroxisome proliferator-activated receptor-α (PPARα). Conversely, genetic ablation of hepatic ATX or neutralization by antibodies effectively alleviated hepatic steatosis, inflammation, and fibrosis in mouse models (36). Given the implicated pathological linkage of PBC with PPARα, it is possible that ATX-LPA signaling activity contributes to PBC progression through the suppression of PPARα (37).

ATX has also been identified as a novel player in the pathogenesis of HCC (28). ATX binds to LPA and adhesion molecules, including integrins, to potentially contribute to the cancer metastasis (38). Along with vascular endothelial growth factor receptor-2 and vascular endothelial growth factor receptor-3, ATX is reportedly involved in vascular development in the liver, which may contribute to HCC onset during the progression of chronic viral hepatitis C (39). Supporting these results, a recent report indicated that serum ATX levels after antiviral therapy for hepatitis C could predict the formation of HCC during long-term follow-up (40). In addition to a role in HCC development, ATX-LPA signaling has been associated with the etiology of other LRE, including esophagogastric varices, ascites, and hepatic encephalopathy. Hepatic encephalopathy is a neurological complication of liver disease that is thought to be caused by the accumulation of ammonia and other toxic substances in the blood. ATX-LPA signaling was shown to increase the production of ammonia in the liver and lead to greater blood-brain barrier permeability (41), which could contribute to hepatic encephalopathy onset (42). In addition, ATX-LPA signaling was found to increase blood vessel permeability in the liver, potentially leading to fluid leakage into the peritoneal cavity and ascites, and raise the production of angiogenic factors promoting angiogenesis toward the varices development (43). Indeed, serum ATX levels were seen to be high in cirrhotic patients with ruptured varices, ascites, and encephalopathy (44). Taken together, ATX-LPA signaling may be involved in several LRE, including HCC, esophagogastric varices, ascites, and hepatic encephalopathy, independently of liver fibrosis. Measuring serum ATX levels might therefore assist in predicting future LRE.

Last, previous research supports a significant gender disparity in reference levels of ATX, with higher values observed in women (21). This difference is believed to be influenced by estrogen, which reportedly regulates ATX production and secretion (45). It is therefore important to consider gender as a significant factor when interpreting patient ATX levels. Despite incorporating gender into the multivariate analysis of our cohort; however, ATX remained independently associated with LRE (Table 4), thus corroborating its relationship with LRE regardless of gender.

### Strengths and limitations

The main strengths of this study lie in its substantial patient cohort, long-term observational design, and validation of results through an additional cohort to ensure robust and reliable findings regarding LRE in PBC. This study also had limitations, including a retrospective design and exclusive focus on Japanese individuals. Future research should validate our findings in more diverse cohorts and monitor serum ATX levels longitudinally to establish its clinical significance in PBC.

### Future implications

ATX holds significant promise as a dependable biomarker for predicting long-term LRE in PBC, such as HCC, esophagogastric varices, ascites, and hepatic encephalopathy. Using ATX as a clinical prognostic tool has the potential to pinpoint high-risk patients, consequently leading to improved treatment and enhanced disease management.

## CONFLICTS OF INTEREST

**Guarantor of the article:** Takefumi Kimura, MD, PhD.

**Specific author contributions:** T.K. and T.I.: designed the research. T.I., T.K., T.O., W.S., H.K., Y.Y., A.S., T.Y., and S.J.: collected clinical data. T.I., S.S., and K.I.: performed the assays. T.I. and T.K.: analyzed the data. T.I. and T.K.: wrote the paper. T.U.: supervised the study.

**Financial support:** This research was supported by AMED under grant number JP23fk0210125, JP24fk0210125 and by JSPS KAKENHI grant number JP22K20884 and JP24K11087 and by the Research Center for GLOBAL and LOCAL Infectious Diseases, Oita University (2024B11).

**Potential competing interests:** S.S. and K.I. are employees of TOSOH Corporation. The remaining authors declare that they have nothing to disclose regarding funding from industries or other conflicts of interest with respect to this manuscript.

**Study Highlights**

**WHAT IS KNOWN**

✓ Primary biliary cholangitis (PBC) is a chronic autoimmune liver disease associated with significant risks of liver-related events (LRE), including hepatocellular carcinoma (HCC), esophagogastric varices, ascites, and hepatic encephalopathy.
✓ Although various predictive markers such as FIB-4, ALBI, APRI, and M2BPGi have been developed, their accuracy in predicting LRE remains suboptimal, highlighting the need for more effective, non-invasive prognostic tools.

**WHAT IS NEW HERE**

✓ This study identifies serum autotaxin (ATX) levels as a robust, independent predictor of LRE in untreated PBC patients, surpassing the predictive capabilities of traditional markers such as FIB-4, ALBI, APRI, and M2BPGi.
✓ With serum ATX levels ≥ 1.086 mg/L showing a significantly elevated cumulative incidence of LRE, and high AUROC values validated in both primary and validation cohorts, ATX emerges as a promising novel biomarker for clinical prognostication in PBC management.

## Supplementary Material
