# Supplementary material for: Serum Autotaxin Levels Predict Liver-Related Events in Patients With Primary Biliary Cholangitis: A Long-Term Multicenter Observational Study
Source: Clin Transl Gastroenterol. 2024 Oct 17;15(12):e00779. doi: 10.14309/ctg.0000000000000779 (PMC11671064; doi:10.14309/ctg.0000000000000779)
Supplement: Supplementary file 1 [file ct9-15-e00779-s001.pptx]

## Slide 1
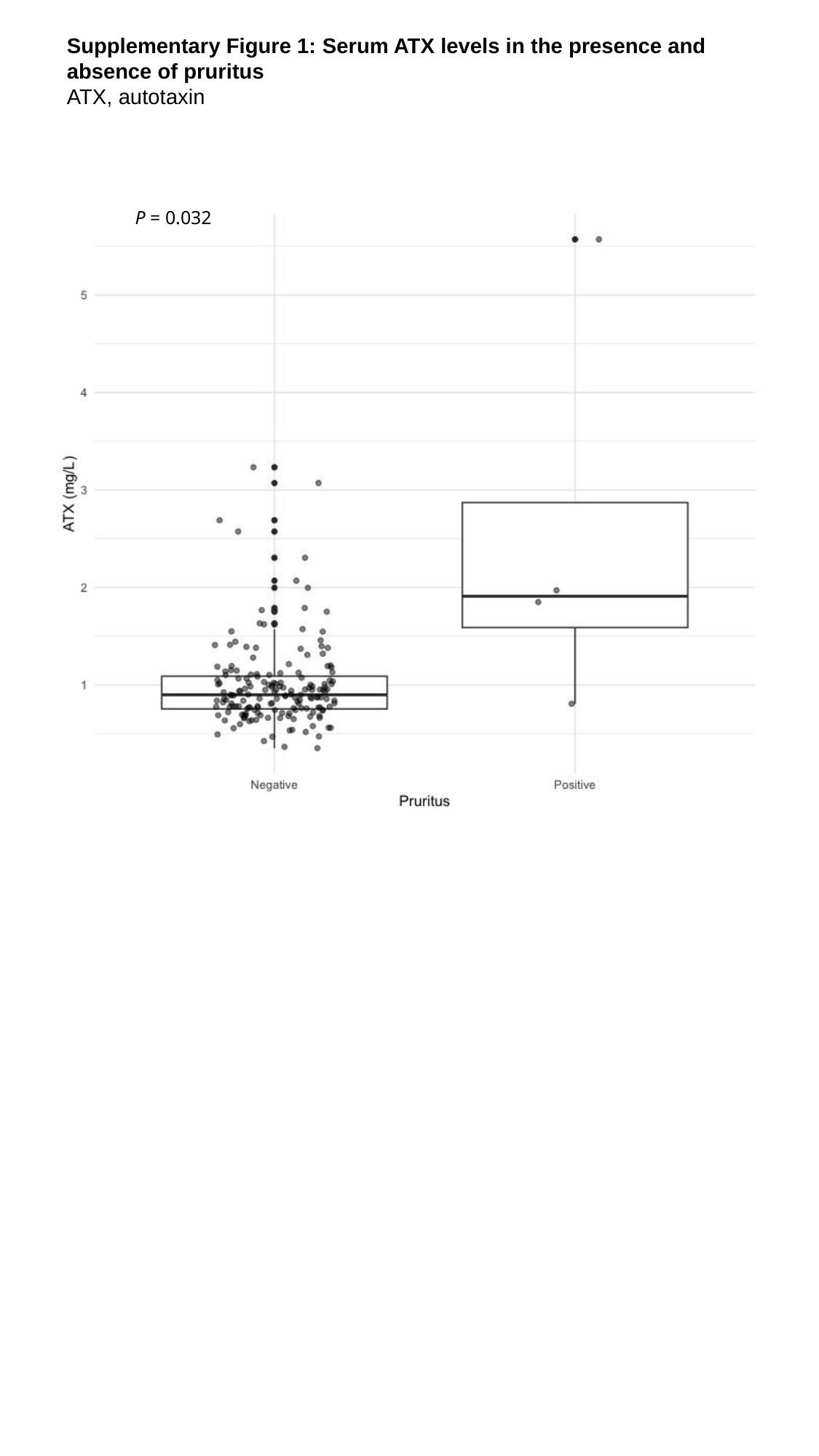

Supplementary Figure 1: Serum ATX levels in the presence and absence of pruritus
ATX, autotaxin
P = 0.032

## Slide 2
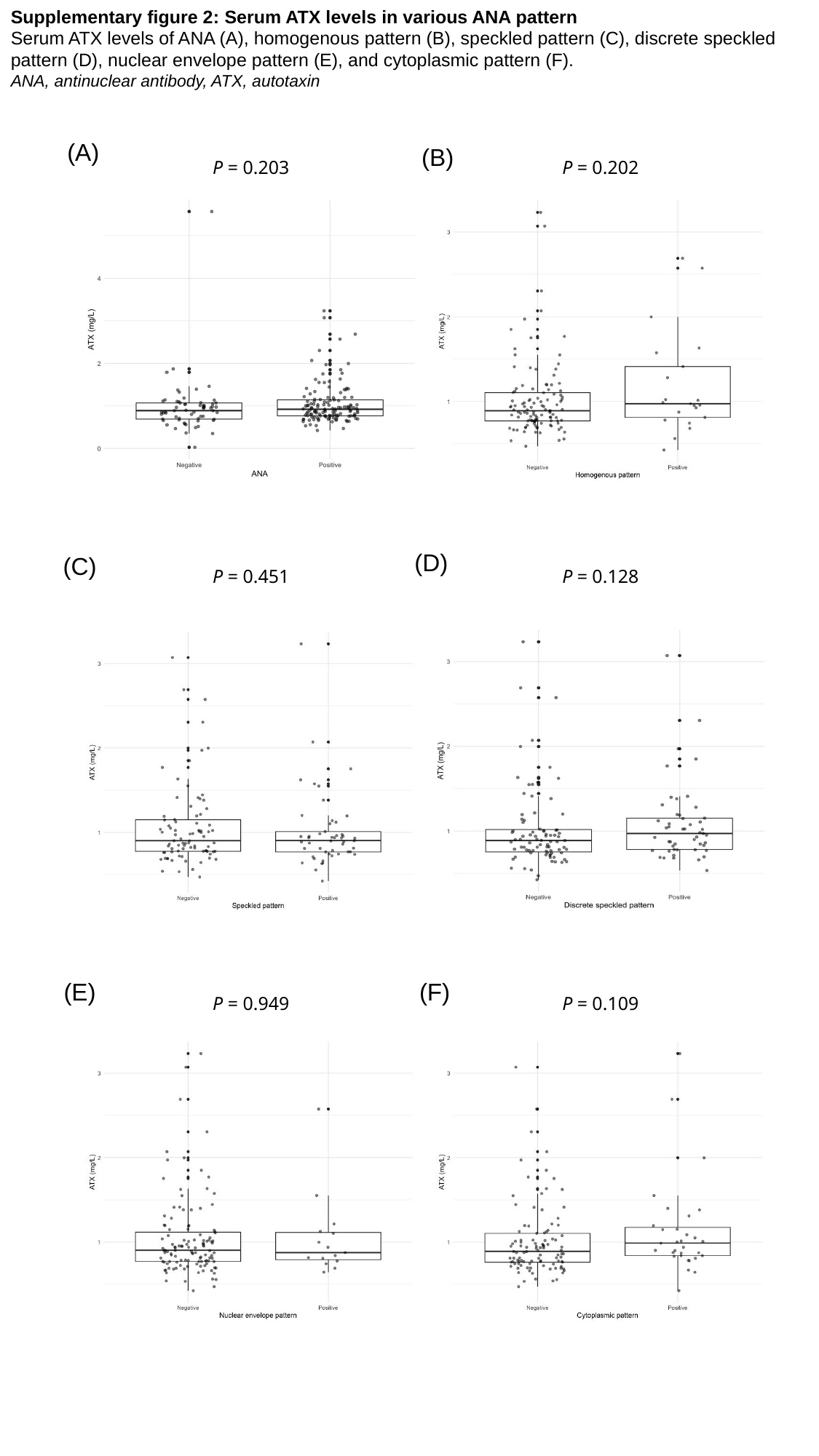

Supplementary figure 2: Serum ATX levels in various ANA pattern
Serum ATX levels of ANA (A), homogenous pattern (B), speckled pattern (C), discrete speckled pattern (D), nuclear envelope pattern (E), and cytoplasmic pattern (F).
ANA, antinuclear antibody, ATX, autotaxin
(A)
(B)
P = 0.203
P = 0.202
(D)
(C)
P = 0.451
P = 0.128
(E)
(F)
P = 0.949
P = 0.109

## Slide 3
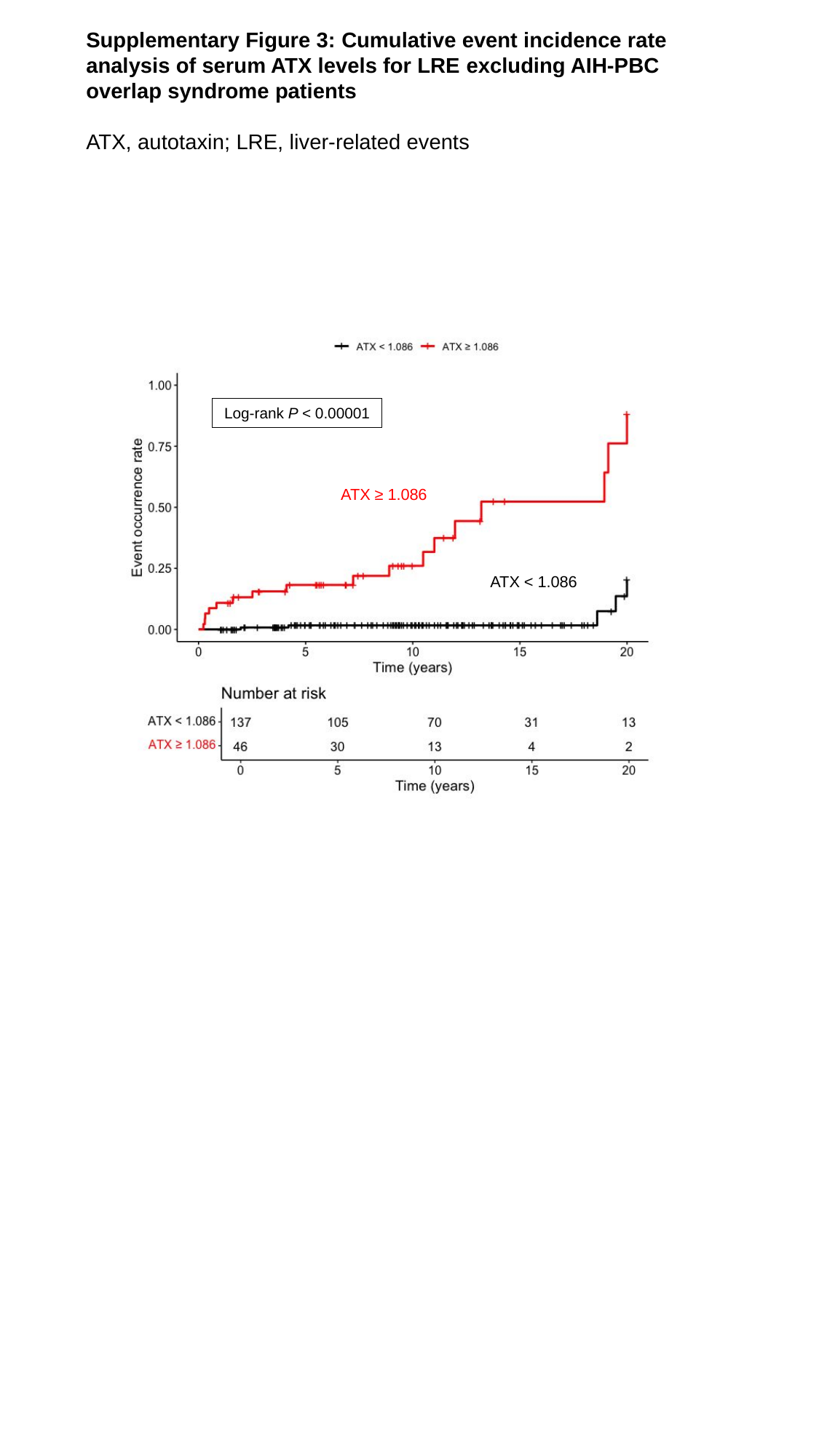

Supplementary Figure 3: Cumulative event incidence rate analysis of serum ATX levels for LRE excluding AIH-PBC overlap syndrome patients
ATX, autotaxin; LRE, liver-related events
Log-rank P < 0.00001
ATX ≥ 1.086
ATX < 1.086
