# Supplementary material for: Serum Autotaxin Levels Predict Liver-Related Events in Patients With Primary Biliary Cholangitis: A Long-Term Multicenter Observational Study
Source: Clin Transl Gastroenterol. 2024 Oct 17;15(12):e00779. doi: 10.14309/ctg.0000000000000779 (PMC11671064; doi:10.14309/ctg.0000000000000779)
Supplement: Supplementary file 2 [file ct9-15-e00779-s002.docx]

**Supplement Table 1. Clinical characteristics of patients with PBC in validation cohort**

|  | **Median (IQR) / n (%)** |
| --- | --- |
| Age (years) | 63 (57-70) |
| Male | 10 (31) |
| Albumin (g/dL) | 4.0 (3.9-4.4) |
| T-bil (mg/dL) | 0.7 (0.5-1.0) |
| AST (U/L) | 35 (27-46) |
| ALT (U/L) | 32 (22-56) |
| ALP (U/L) | 124 (96-169) |
| γ-GT (U/L) | 111 (56-189) |
| Plt (×10^4^/μL) | 21.5 (15.4-24.7) |
| ATX (mg/L) | 0.81 (0.73-1.08) |

ALT, alanine aminotransferase; ALP, alkaline phosphatase; AST, aspartate aminotransferase; ATX, autotaxin; γ-GT, gamma-glutamyltransferase; IQR, interquartile range; PBC, primary biliary cholangitis; Plt, platelet count; T-bil, total bilirubin

**Supplement Table 2. Events during follow-up in validation cohort**

|  | All (n = 32) |
| --- | --- |
|  | Median (IQR) / n (%) |
| Follow-up (years) | 5.3 (3.1-10.3) |
| **Events during follow-up** |  |
| LRE^＊^ | 4 (12.5) |
| HCC | 1 (3.1) |
| Esophagogastric varices | 2 (6.3) |
| Ascites | 2 (6.3) |
| Hepatic encephalopathy | 2 (6.3) |

＊Including 2 patients in whom multiple LRE were found simultaneously: 1 case of varices ＋ ascites, 1 case of HCC ＋ ascites ＋ encephalopathy. HCC, hepatocellular carcinoma; IQR, interquartile range; LRE, liver-related events

**Supplement Table 3. Comparison of clinicopathological features at time of biopsy between non-LRE and LRE patients in validation cohort**

|  | **Non-LRE (n = 28)** | **LRE (n = 4)** |  |
| --- | --- | --- | --- |
|  | Median (IQR) / n (%) | Median (IQR) / n (%) | p value |
| Age (years) | 63 (56-70) | 64 (57-73) | 0.588 |
| Male | 10 (36) | 1 (25) | 0.678 |
| Albumin (g/dL) | 4.0 (3.9-4.3) | 4.0 (3.5-4.1) | 0.341 |
| T-bil (mg/dL) | 0.7 (0.5-0.9) | 1.0 (0.6-1.4) | 0.190 |
| AST (U/L) | 33 (25-44) | 51 (39-69) | 0.082 |
| ALT (U/L) | 31 (21-62) | 40 (28-53) | 0.569 |
| ALP (U/L) | 119 (95-167) | 169 (104-348) | 0.060 |
| γ-GT (U/L) | 109 (54-192) | 183 (102-468) | 0.231 |
| Plt (×10^4^/μL) | 21.9 (16.1-24.8) | 13.0 (10.2-24.8) | 0.210 |
| ATX (mg/L) | 0.78 (0.72-1.02) | 1.68 (1.55-1.85) | **0.007** |

ALT, alanine aminotransferase; ALP, alkaline phosphatase; AST, aspartate aminotransferase; ATX, autotaxin; γ-GT, gamma-glutamyltransferase; IQR, interquartile range; LRE, liver-related events; Plt, platelet count; T-bil, total bilirubin

**Supplement Table 4. Factors associated with LRE in PBC in univariate Cox proportional hazards testing**

|  | **Univariate** | | | |
| --- | --- | --- | --- | --- |
|  | **HR** | **95% CI of HR** | **p value** | **c-index** |
| **Age**  **(≥ 65 years)** | 1.92 | 0.76-4.86 | 0.200 | 0.573 |
| **Gender**  **(Female)** | 0.56 | 0.19-1.71 | 0.300 | 0.549 |
| **FIB-4**  **(≥ 3.42)** | 7.73 | 3.20-18.68 | **< 0.001** | 0.723 |
| **ALBI**  **(≥ -2.492)** | 6.38 | 2.72-14.97 | **< 0.001** | 0.741 |
| **M2BPGi**  **(≥ 1.21)** | 9.77 | 2.96-32.12 | **< 0.001** | 0.762 |
| **APRI**  **(≥ 0.71)** | 6.49 | 2.64-15.93 | **< 0.001** | 0.788 |
| **Nakanuma**  **stage** | 4.27 | 2.62-6.97 | **< 0.001** | 0.778 |
| **ATX**  **(≥ 1.086)** | 12.56 | 4.61-34.21 | **< 0.001** | 0.806 |

ALBI, albumin-bilirubin score; APRI, aspartate aminotransferase to platelet ratio index; ATX, autotaxin; CI, confidence interval; FIB-4, fibrosis-4 index; HR, hazard ratio; LRE, liver-related events; M2BPGi, Mac-2-binding protein glycan isomer; PBC, primary biliary cholangitis
